# DEALING WITH DISASTER DATABASES – WHAT CAN WE LEARN FROM HEALTH AND SYSTEMATIC REVIEWS? Application in practice

**DOI:** 10.1371/currents.RRN1272

**Published:** 2011-09-30

**Authors:** Ishani Kar-Purkayastha, Mike Clarke, Virginia Murray

**Affiliations:** ^*^Specialist Registrar in Public Health & Clinical Toxicology, Centre for Radiation, Chemicals and Environmental Hazards, Health Protection Agency; ^†^Director, All Ireland Hub for Trials Methodology Research; Evidence Aid and ^‡^Head of Extreme Events and Health Protection Centre for Radiation, Chemicals and Environmental Hazards, Health Protection Agency, London UK

## Abstract

There is an increasing move towards facilitating the use of research findings in policy and practice relating to disaster risk reduction and response. One of the key issues is the quality of the evidence available to decision-makers. Disaster databases, as a key resource, represent a tremendous investment of effort and goodwill. However, their usefulness is limited by the variability in how they are compiled, differences in the output they produce, a general lack of comparability and standardization, and the fact that they might produce different results due to the ways they have been created or by chance. One possible solution to this, which has been applied successfully in evidence synthesis in health care is the systematic review. In this study we attempt to show how the systematic review process may be applied to information and data that is held in disaster databases. We demonstrate that systematic reviews of disaster databases can be achieved in a technical sense and the potential value of such reviews, but also discuss the practical difficulties that arise.

Key words

Systematic review, evidence, disaster database

**Introduction**

The international risk and disaster community appears to be faced with an increasing number of extreme events, with ever greater numbers of people being affected as a consequence [1].  Moreover, the shift in emphasis within disaster management towards planning and preparedness has exacerbated the need for reliable data on disaster impacts to inform policy-makers and practitioners at all stages. Reliable knowledge about what happened in past disasters may help in the planning for the future.

However, it is often difficult to establish reliably the scale and impact of natural disasters. This is in part due to the large variety of potential data sources available, which can be difficult to navigate effectively and the differences between these sources. This has produced a voluminous yet inconsistent evidence base for policy-makers and practitioners in the field to draw upon and runs the risk of undue emphasis on the output from a single source. 

Some of the inconsistencies are due to shortcomings in the disaster data itself and include lack of standardisation in collection methodologies and definitions, as well as the absence of a single reliable source of verified data [2]. Moreover, the databases themselves are hosted by a variety of organisations, with different disciplinary affiliations and scientific traditions. Individual databases are usually set up with distinct objectives, which may be inconsistent with those of other databases. It is not surprising, therefore, that it is often difficult to compare outputs across databases and this has been found in previous comparisons of disaster databases [3] [4]. However, this lack of shared focus makes it difficult to come to a consensus on the range and magnitude of disaster impacts and, as a result, to have confidence in the estimates presented. 

Recognizing some of these issues, the United Nations International Strategy for Disaster Reduction (UNISDR) identified the need for a closer interface between science and policy at the second session of the Global Platform for Disaster Risk Reduction [5]. In 2011, the Science and Technical Committee of the UNISDR re-iterated the ongoing need for review and consolidation of the various disaster information sources and databases, in order to improve the consistency, comparability, quality assurance and value of the data available to the international risk and disaster community.  

Challenges in ‘managing’ data or evidence are not exclusive to the field of disasters. In healthcare, for instance, the volume of research can be overwhelming – as an illustration – the world’s largest repository of reports of studies of healthcare interventions, the Cochrane Central Register of Controlled Trials (CENTRAL), now contains more than 600,000 records and published reports of trials are increasing at the rate of more than 25,000 per year. Similarly, the World Health Organisation (WHO) hosts the International Clinical Trials Registry Platform (ICTRP). Considerable increases are also apparent in the number of reports of other types of health related research. 

One of the solutions to this problem in health care has been the increasingly common conduct of systematic reviews, led by the work of The Cochrane Collaboration [6], but with many others now also working on the more than 2500 systematic reviews produced each year [7].   

**The aim of this paper**

In the context of the overarching aim of the UNISDR to augment the role of science in disaster risk reduction, and the need for effective management of disaster data, this paper investigates how the systematic review approach might be used to overcome the challenges of using the disaster databases to inform decision making. In presenting the systematic review as a method for managing data or evidence we draw on previous explanations of the systematic review as a method of locating, appraising and synthesising evidence following the formulation of a clearly focused question. This helps to maximise the power of the previous research, avoiding undue emphasis on any single source or opinion, and doing so in a transparent way, which allows users of the review to see and, if they wish, critique the methods adopted.  Simply, we aim to demonstrate the applicability of the systematic review as a tool to critically appraise the quality of the data presented in the disaster databases and, if appropriate, to combine their findings to produce a summary estimate which is as unbiased as possible.   

Whereas authors in the past have undertaken evaluations of disaster databases against set criteria [3] [4], our aim in using the systematic review was to orientate our evaluation to the perspective of a policy-maker or practitioner with specific questions to answer. Different databases may be set up with different objectives in mind, some of which may be competing e.g. speed or precision. Rather than evaluating how well a database meets the objectives it was set up for, our approach seeks to evaluate how well each database or databases collectively are able to meet the information needs of policy-makers or practitioners by interrogating the databases with specific practice-orientated questions in mind.

**Evidence in health – the current role of systematic reviews**

*Types of evidence* 

Decision-making in health care uses different types of evidence for resolving uncertainties about, for example, effectiveness and cost effectiveness (of an intervention or a programme), need (e.g. to provide a profile of disease burden in a population), epidemiology and risk factors (for particular illnesses or conditions) and user acceptability (of an intervention or means of delivering a service).   

*Sources of evidence*

The most appropriate source of evidence depends very much on the type of evidence required. For instance, where the effectiveness of an intervention is being considered, the evidence will typically come from research using interventional study designs, such as randomized trials. Whereas, evidence of need may more often come from observational studies, and evidence of acceptability or patient experience is most likely to come from qualitative research.   

There are various schemes by which the relevance and value of different ‘sources’ of evidence used in health care are ranked. The hierarchy developed by the Centre for Evidence-Based Medicine at Oxford (CEBM) [8], is very much grounded in the bio-medical tradition of experimental research, building from expert opinion, through case series, case-control studies, cohort studies and randomized trials, to culminate in systematic reviews. However, this hierarchy is not suited to all types of evidence and is not universally appropriate or practicable, for example, in public health where the question being posed may be about prognosis following an environmental exposure, in clinical policy research, or in exploring patient experiences. Alternative schemes for grading evidence have been proposed for use in these and other contexts [9]. However, there is general agreement around the usefulness and position of systematic reviews within each hierarchy, in that this form of research should be at or near the top.   

*The role of systematic reviews* 

Systematic reviews help people to overcome the ever increasing “information overload”, by providing summaries of the body of research relevant to a particular topic. Their structured approach, transparency of methods and detailed reporting also allow systematic reviews to minimise the impact that bias might have on their findings and conclusions.   

Systematic reviews also provide a means by which the quality of the constituent studies can be appraised in a standard way, allowing the authors to compare, contrast and, if appropriate, combine the findings of the individual studies to provide an overall summary of the topic being investigated, be it the reduction in blood pressure following an anti-hypertensive drug, the increased incidence of cancer following radiation exposure, or the number of people who are likely to develop pressure ulcers while in hospital. Systematic reviews differ from traditional review articles in that they avoid selective emphasis on individual studies or reliance on the personal knowledge of research by the authors of the review.   

Systematic reviews are a recognised form of research and involve a well-developed series of steps to minimise bias and to maximise their relevance and reliability [10]. Key steps include the formulation of a clear question and the associated eligibility criteria for the review, a rigorous and wide-ranging search for the evidence, its appraisal, synthesis and summary (including statistical analyses, or meta-analyses, where appropriate), and the interpretation of the findings, which might include implications for practice [7] [11] [12]. As a result, systematic reviews have become a key bridge between individual research studies and decision-making in healthcare [13]. Although the majority of systematic reviews in health care relate to questions about the effectiveness of interventions, many also exist in other areas noted above [7].   

Another key outcome of the systematic review process is that it can highlight an absence of evidence and suggest areas for further research.   

**Evidence in disasters – the proposed role for systematic reviews**

*Types of evidence*

The disaster community is pluralistic with a range of disciplines contributing, with different traditions of enquiry, different epistemological leanings, languages and tools. From a health sector perspective, the types of evidence that are useful to practitioners and policy-makers in planning for and responding to disasters is the same as the types of evidence they would find useful in any other setting.  

*Sources of evidence* 

The relative contributions, however, from different sources to the overall evidence base is quite different to the non-disaster setting. This is in part a result of feasibility, with interventional studies such as randomized trials often felt to be impossible, unethical or, simply, not feasible [14] [15] [16]. In such circumstances, evidence on the relative effectiveness of different interventions to guide policy and practice will usually need to come from systematic reviews of research in non-disaster settings. Some efforts are already underway to bring together these resources, so that practitioners can more easily access this knowledge and use it to inform practice in the field. For example, Evidence Aid [17] began as an initiative by The Cochrane Collaboration to provide summaries of evidence on the effects of healthcare interventions for use by the disaster community. Although this is able, to some extent, to draw on the thousands of systematic reviews that exist in health care, other areas of disaster planning and response lack such a large and comprehensive body of systematic reviews. For example, areas such as engineering, communication, shelter and security do not have resources such as those created in health. 

There is also a need for the other types of evidence mentioned above, including evidence of need, which may come through observational epidemiological studies, field data or needs assessments [18], and evidence of impact and consequences, which is also most likely to come from observational epidemiology in the post-disaster setting. As with the need for evidence on the effects of interventions, the requirement for other types of evidence exists in health, and also across all of the other disciplines that make up the disaster community.  

*The role of disaster databases: contribution to the evidence base* 

There are various definitions of the term “database” [19] [20] [21] (but, at a minimum, it is a collection of data, organized with a regular structure that, today, is usually in an electronic format). The disaster databases include information on, for example, the number of people affected, damage to property and infrastructure and the use of resources. However, the scope of what is collected, how it is presented, the validation processes used, accessibility and other such features of the database depend upon the people responsible for the database, and in turn upon their objectives when creating the databases. 

**Systematic review of disaster databases**

Disaster databases record outcomes such as ‘number of people affected’ or ‘economic losses’. These data might include absolute numbers or frequencies – incidence or prevalence – and are, therefore, similar to the types of information gathered in other health contexts. Estimates of absolute numbers or of frequency may come from observational epidemiological studies; or through case registration in clinical databases or disease registries (such as cancer registries or registers of congenital anomalies). Therefore, the format and concept for the disaster databases most closely resemble clinical databases or disease registries and they would be placed alongside other observational methods in the traditional bio-medical evidence hierarchies. This makes them amenable to the systematic review process, and the process has previously been applied to disease registers in other contexts [22]. The key steps of a systematic review are: (i) the systematic identification of all evidence relevant to the question being posed; (ii) critical appraisal of all sources and (iii) synthesis of a summary. 

The primary objective of the systematic review is to provide a summary of the evidence relating to a particular question by combining evidence from multiple sources; systematic reviews may also identify a lack of consistency and be used to explore the sources of any heterogeneity. The database is evaluated in the context of its usefulness in answering the question posed. 

The questions that might be addressed through a systematic review of the disaster databases include health-related issues such as burden of infectious disease or psychological illness following a disaster, but systematic reviews of the databases could be applied within any domain. Examples of non-health related questions in the disaster setting might be how many bridges were destroyed or how many schools need to be rebuilt.   

The step-wise process for applying systematic review methods to a disaster related question can be illustrated by the following outline. This follows the structure used for Cochrane reviews [11], which are widely regarded as the gold standard for systematic reviews in health care.   

The first step is to choose and state a clear question for the review. This might be broad, such as “How many people are affected by disasters?” Alternatively the question may be narrowed down to “How many people will develop gastrointestinal disease following a disaster?” Or even further to “How many young children will develop gastrointestinal disease in the four-week period following flooding in affected regions in low-income countries?” Defining the question sets the scope for the systematic review, and this can then be used to create a structure for the eligibility criteria for the review, which can be categorized as follows: (1) the types of participants (for example, children aged between 3 months and 5 years); (2) the types of impact (flooding in low-income countries); (3) outcome measures of interest (gastrointestinal disease within 4 weeks of the onset of flooding) and (4) the types of database to use (for example those with a focus on health-related outcomes and a reliable means for gathering data on illness in children). The review should then seek to be as comprehensive as possible in identifying relevant evidence. This should include compilation of a list of all disaster databases that are currently available, which contain data that are relevant to the question being asked. Resources such as the disaster data portal (DisDAT) [23], which indexes 66 registered databases: 15 global; 43 national; 2 supra-national and 4 sub-national) may help with this. These searches might be supplemented by searches using internet search engines and by access to internal databases such as those within Ministries of Health, hospitals, etc.   

Having identified the potentially eligible sources of evidence for the systematic review, the quality of these sources is assessed. For the disaster databases, this might include consideration of the precision of the data, an evaluation of which would include

- Outcome figures (i.e. extent of rounding or the provision of absolute counts)
- Outcome resolution (e.g. disaggregated reporting of outcomes such as “number of gastrointestinal diseases cases” which might be the number people affected or the number of separate episodes)
- Geographical resolution (region, country, town, section of town, family unit)
- Temporal resolution (year, month, week, day)

The accuracy of the data should also be considered, along with its timeliness (i.e. whether all the relevant data have been gathered and included in the database, and whether some data are still being processed) and reliability (i.e. the quality of the sources of the data and the methods used to arrive at any approximations.)

The methods for the review will also need to describe how data will be extracted from the databases.  This might rely on integrated search tools, but, even so, the interrogation terms used should be explicitly stated (including the use of any drop-down menus if these exist within the particular database) and any assumptions made in extracting the data need to be described clearly. 

The analysis plan for the review should include details of the types of statistical analyses performed, along with information on any subgroup or sensitivity analyses (for example, analysis focused on data from databases that were assessed to be of the highest quality).  

The findings of this quality assessment, extraction and analyses should be presented clearly in the systematic review. The contribution of each database and any other sources of evidence should be described, so that readers can see where the evidence comes from, and the numerical basis of any analyses should also be transparent. The conclusions of the review should address the applicability of the findings to other scenarios or situations by identifying contextual characteristics of the included data, for example, the area or people affected, which may support the use of the findings when planning for, or responding to, similar events in other settings which share contextual features.

**Two case studies – exploratory systematic reviews using disaster databases**

To illustrate how systematic reviews might be used in the analysis of disaster databases, we undertook two abridged systematic reviews as case studies. The rationale for undertaking abridged, rather than complete, systematic reviews was the time required. Since our main purpose was to demonstrate proof of concept (i.e. the applicability and feasibility of systematic review to disaster databases as a method), rather than seeking to reach conclusive answers to either of the questions posed as case studies, the time needed to conduct a full review was not justified.

**Case study 1: What was the level of homelessness due to the earthquake in Pakistan in 2005?**

Information on the human and economic costs of natural disasters, such as earthquakes, is critical in informing the immediate response as well as the process for long-term recovery, and future preparedness. It is particularly useful when the data are broken apart to allow an understanding of how people are affected by, for example, trauma, homelessness and psychosocial effects. This enables responders to tailor the delivery of resources according to need and may be used to guide response in future events. Therefore, we sought to examine one aspect of the consequences of the earthquake in Pakistan in 2005: the number of people left homeless in that country. We defined homeless as people whose homes were destroyed and who required alternative accommodation and limited this to those who were made homeless as an immediate and direct consequence of the earthquake. We did not wish the estimate to include people who were homeless before the event.  

We set the following eligibility criteria for the review:

- Population: people living in Pakistan
- Event: earthquake on October 8, 2005 in northern Pakistan
- Outcome measures: homelessness, defined as those who required alternative accommodation (be it state organised or with family or friends) within 48 hours of the earthquake. People leaving their homes for family and community reasons, as well as for safety reasons, are eligible. People who were homeless before the earthquake are not eligible. We planned to perform subgroup analyses by age (<5, 6-18, 19-65 and >65 years) and gender (male, female).
- Databases: were included if available content related to the relevant time period (2005), type of disaster (earthquakes), geographical area (Pakistan) and outcome (homelessness).

We used the search engine on the Disaster Data Portal (DisDAT) to identify suitable databases from amongst its 66 indexed databases. The search limits used in DisDAT were selected from the integrated drop down menus, and are restricted to one of the following four criteria at a time:

- geo-coverage classification (categories include global, national, supra-national, sub-national)
- national coverage country
- type of event (categories include natural, technological, both)
- natural event

It is not possible to search using search parameters for time period or outcome.

If we had progressed to a full review, we would have also conducted searches for potentially eligible databases through internet search engines, such as Google; searches of electronic, bibliographic databases including EM-BIB, MEDLINE, EMBASE, and Web of Science for literature to supplement the database review; and consultation with experts in this area.   

The potentially eligible databases identified in DisDAT were assessed by one author (IK-P) according to the following criteria.

- Access to the database:       Publicly accessible, online or offline
- Precision of data:
  - Counts (rounding to the nearest hundred was acceptable)
  - Outcome (homelessness had to be defined)
  - Geography (at the level of the country: Pakistan)
  - Time (homelessness counted within 48 hours of the earthquake)
- Accuracy of the data       Process used to validate data
- Timeliness (This was not anticipated to be a problem, because more than three years had passed since the earthquake)
- Reliability
  - Sources of the data
  - Quality of these sources
  - Methods used for estimates

The search of DisDAT identified nine potentially eligible databases: The Disaster Database Project, EM-DAT, SIGMA (SwissRe), ADRC Disaster Information for Member Countries, USGS Earthquake Database (USGS), The NGCD Natural Hazard Data, The NGCD Significant Earthquake Database, GLIDE and NGCD Natural Hazards Data Resource Directory. However, detailed assessment of these revealed that it was not possible to use them to provide a reliable answer to our question about the number of people made homeless by the Pakistan earthquake. None of the databases met the inclusion criteria for this review. There was a lack of good quality data, insufficient information on the sources of the data and how it had been collected, lack of transparency for the reporting methods, uncertainty about data validation, poor consistency across the estimates that were available and insufficient detail for the proposed sub-group analyses. Three databases did report the number of homeless people, and the values reported were 5 million, 3.3 million and a range of 2.8 to 3.3 million. However, there was no indication of the level of homelessness in the region before the earthquake and there was insufficient information to identify the number made homeless within 48 hours of the earthquake. A fourth database put the number of homes destroyed in the range 101 to 1000 which, if we were to assume 10 people per household, would equate to only 10,000 people. 

**Case study 2:  Burden of ill-health due to gastrointestinal illness caused by flooding in India during 1999 to 2008**

This systematic review set out to summarise the evidence on the burden of ill-health caused by gastrointestinal illness following flooding events in India over ten years (1999 to 2008). This would be useful for planning the deployment of healthcare resources in the event of future flooding. The objective was to assess how many people in India experienced symptoms of gastrointestinal illness (vomiting and diarrhoea only) or had a diagnosis of gastrointestinal illness as a result of flooding events. We defined gastrointestinal illness as a diagnosis by a clinician of gastrointestinal illness, or someone reporting symptoms of diarrhoea or vomiting, and wished to include only those cases that had been caused by flooding.  

We set the following eligibility criteria for the review:

- Population: people living in India
- Event: compromised drinking water as a result of a flooding event, excluding sources of drinking water that were contaminated before the flooding.
- Outcome measures: gastrointestinal illness with symptoms of vomiting or diarrhoea, including specific diseases such as hepatitis, cholera or typhoid. We planned to perform subgroup analyses by age (<5, 6-18, 19-65 and >65 years) and gender (male, female).
- Databases: include the relevant time period (1999 to 2008), type of disaster (flooding), geographical area (India) and outcome (gastrointestinal illness).

We searched DisDAT and identified two databases with coverage of India, and 47 (including those two) for flooding. A preliminary search using the internet search engine Google identified 110,000 pages using the key words “disaster database India flood gastrointestinal”, in October 2009 but these hits were not assessed. We contacted experts in the area for suggestions for additional databases. As with the first case study, a full systematic review would have also included searches of electronic, bibliographic databases including EM-BIB, MEDLINE, EMBASE and Web of Science.  

The potentially eligible databases identified in DisDAT were assessed by one author (IK-P) according to the following criteria.

- Access to the database:       Publicly accessible, online or offline
- Precision of data:
  - Counts (rounding to the nearest hundred was acceptable)
  - Outcome (gastrointestinal illness, vomiting or diarrhoea had to be defined)
  - Geography (at the level of regions of India or the country as a whole)
  - Time (gastrointestinal illness within a specified time of a flooding event)
- Accuracy of the data
  - Process used to validate data
  - Adjustments to take account of baseline levels of gastrointestinal illness
- Timeliness (This might be a problem if the compilation of the database for the recent years in 1999 to 2008 is not sufficiently up to date)
- Reliability
  - Sources of the data
  - Quality of these sources
  - Methods used for estimates

The search of DisDAT identified eight potentially eligible databases: The Disaster Database Project, EM-DAT, SIGMA (SwissRe), ADRC Disaster Information for Member Countries, India national disaster database – InDisDATA, The Dartmouth Flood Observatory (DFO) database, NGCD Natural Hazards Data Resource Directory and Orissa, India database – DesInventar (duplicate). The experts who were contacted suggested two more: GDACS and ReliefWeb. However, as with the abridged systematic review of homelessness following the Pakistan earthquake, assessment of the databases revealed that it was not possible to use them to provide a reliable estimate of the number of people having gastrointestinal illness following flooding events in India in the ten years from 1999. The problems with the databases were similar but were compounded by incomplete information across the time period of interest. Furthermore, we were not able to find data in any of the databases on the number of people affected by gastrointestinal illness following a flooding event in India. This was reported by some media on an ad hoc basis, such as a BBC report of 120 deaths due to leptospirosis in 2005 and the deaths of 76 people in Bihar in 2002 due to water-borne illness, but these numbers are large underestimates of the morbidity and mortality caused by gastrointestinal illness in a decade of flooding in India.   

**Discussion**

Systematic reviews have helped to make the vast amount of research evidence in health care more manageable to policy makers, practitioners, patients and the public and this is also becoming the case in the social sciences [13] [24]. The reviews also provide a means for minimizing bias in the interpretation of findings from individual studies, avoiding undue emphasis on individual studies [25] and improving the design of future research. We believe that these benefits could also be achieved within disaster planning, and that the application of systematic reviews to disaster databases is feasible and would enhance their contribution to the evidence base.   

Although the main applied benefit of systematic reviews is their ability to synthesise and summarise evidence across disparate sources, the methodology has value in itself on account of the procedural rigour used to minimise bias and to critically appraise the included research.   

An important difference between evaluation of databases using measures of concordance or against specified criteria and the systematic review approach is that, in the latter, the critical appraisal of the evidence is focused around questions of relevance to practitioners and policy-makers, rather than on the database or databases *per se*. Therefore, implicit within the process of systematic review is an assessment of the practical utility of current evidence to the needs of key stakeholders. Consequently, even when the results of a systematic review are inconclusive, gaps will have been identified to inform future research, in a procedurally rigorous way which adds to weight to calls for further research in those specific areas.  

That said, application of systematic review to disaster databases whilst technically straightforward, may in practice prove to be problematic. There is a wealth of information available in disaster databases; but, the content – extent and quality – of the data collected depends very much on the respective objectives of the producers of these databases; the methods used for data collection and case definitions, which may vary depending on disciplinary affiliations; and the quality and timeliness of the data, which may vary depending on resources and the interests of the producers. For example, if the objective is to provide a rough estimate quickly to allow prompt action in the early hours of disasters, this will be of less value to a systematic review that seeks a more validated and accurate estimate of an impact at a much later date. These and other characteristics of the databases themselves can limit comparability and provide examples of the types of heterogeneity that should be explored within a systematic review.   

A second, perhaps more fundamental issue lies with the quality and relevance of the primary data being collected and made available within the databases. Databases, like any secondary source of information, are limited by the primary data on which they draw. The difficulties surrounding the collection of primary data have been commented on previously and include lack of standardized case definitions, difficulty defining population denominators, attributing causality, and lack of comparability between sources, to name just a few [2] [26] [27]. At the same time, there is often a large amount of activity in the aftermath of a disaster, with many agencies intervening and collecting data for their own internal use. Data collection requires resource and, particularly in resource-stretched settings such as in the aftermath of a disaster, there is an ethical imperative to ensure that all data collected is of good quality, and is useful and relevant to as many users as possible.  

The challenges we encountered in using the disaster databases as a source of evidence for these two abridged systematic reviews are akin to those faced by reviewers of clinical trials in health care in the 1980s. The raw material for their work - the reports of the original research - did not contain sufficient information. There were shortcomings in descriptions of the methods of the trials, in the reporting of the results, and in the comprehensiveness of those results. There was a potentially high risk of bias due to how the data had been collected and reported, and the details to assess this risk were not available because of incomplete reporting. Some of these problems persist in health care but they are being tackled by initiatives such as the CONSORT reporting guidelines for randomised trials [28]. Similar work is also being done for other types of study, including observational studies (STROBE) [29], diagnostic studies (STARD) [30], qualitative research (COREQ) [31] and systematic reviews themselves (PRISMA) [12]. There is accumulating evidence that these initiatives have led to improvements in the quality of reporting of research [32].

There are also ongoing efforts in health care to establish consensus and standardise the ways that outcomes are measured, collected and reported in research. The aim being to reach agreement on a minimum set of standard outcomes that would be collected in all relevant research. Some existing examples of this are in rheumatology [33], maternity care [34] and child health [35]. These “core outcome sets” will make it easier to compare, contrast and combine the findings of research in systematic reviews.

Although efforts are underway within the disaster community to standardize data collection, without firm international consensus on priorities and methods, the creation of a common core dataset, which in turn may facilitate cross-validation between sources and greater data credibility, remains a nebulous goal. 

We support the call for a common approach to data management in disasters and propose the systematic review as a method for critically summarizing evidence, but perhaps more crucially, given the findings from our case studies, as a tool by which to identify gaps in the data in a procedurally rigorous and objective way, which may help focus international efforts to fill these gaps and resolve the accompanying uncertainties.  

In order to maximise the usefulness of disaster databases, we suggest a much greater focus on the information needs of policy-makers and practitioners in the disaster community, possibly with database set-up being informed directly by regular evaluation of these information needs. In the disaster context, where there is still so much heterogeneity in the data, perhaps the most pragmatic and instrumental way in which systematic reviews can be used would be to navigate these heterogeneities and through a process of iteration and negotiation between different stakeholders help in the development of a common core dataset. Finally, without buy-in from partners, no data set, however well constructed will not succeed. CONSORT, STROBE and other reporting standards in health were developed by large mullti-party international working groups which developed a consensus that was then accepted widely. Perhaps, a similar process is needed to ensure that candidate core datasets actually achieve the universal (or at least widespread) usage needed in order to make any difference to data quality.   

**Conclusions**

In this paper, we have shown how the systematic review process may be applied to disaster databases to strengthen the role that they can play in planning for, and responding to, disasters. We outline the benefits of doing so and discuss some of the obstacles that we anticipate. We demonstrate proof of concept by applying systematic review methods within two ‘case studies’ and recommend that the role of systematic reviews as a source of reliable information for policy makers and practitioners should be recognized as strongly across the disaster community as it has been in health care. Bearing in mind the current variability across the disaster databases, we also recommend that reporting standards should be established for disaster databases to make it easier for users to identify the particular advantages and disadvantages of each database with regard to specific policy or practice questions and suggest carrying out systematic reviews as an integral part of this development process. If a standardized, minimum dataset could be agreed for routine collection and storage in all databases, this would also go a long way towards increasing the value of these databases for decision makers.   

Analogous to the experience of developing reporting guidelines for clinical studies, the development and acceptance of a core outcome set or sets in the disaster field is likely to be achieved only through an iterative process which will require international co-operation and consensus. Efforts in this direction are already underway [36]. Systematic reviews should also be able to help because in addition to their well recognized role as a process for critical review of existing scientific evidence with the objective of arriving at a concise summary, they are also useful for the systematic identification of knowledge gaps. Given the findings from our case studies, we propose that this benefit of the systematic review will, at least at the outset, be of greatest use in defining a core outcome set for disaster data, as the gaps will have been identified through a transparent, reproducible, objective process and not predicated on the experiences of individuals or organisations. In the longer term, we expect that the strength of systematic reviews as a means of providing answers to key questions for disaster risk reduction and response will come to the fore, as the quality of disaster data improves.   

The disaster databases represent a tremendous investment of resources, effort and good will. At the moment, however, the collective resource of these databases is not realizing its full potential to influence policy and practice for the better. The suggestions outlined in this paper would harness the power of the databases by valuing their diversity and recognizing their different roles whilst at the same time making it easier for users to synthesize evidence from multiple sources and having assurance in choosing the ones most appropriate to their purposes. This, in turn, would help resolve the uncertainties of policy makers, organisations, practitioners and the public, and would lead to reductions in the damaging effects of disasters on populations and societies. The disaster community has an opportunity to benefit from many decades of experience of the conduct of systematic reviews and the implementation of evidence based health care. We hope that this paper will stimulate discussion and the actions needed to grasp this opportunity.

ACKNOWLEDGEMENTS

The authors would like to thank the help and advice provided by the sub-committee on databases of the UNISDR Science and Technical Committee, specifically Dr Walter Ammann; Prof Gordon McBean; Prof Moshen Ghafory-Ashtiany; Prof Laban Ogallo; Dr Kaoru Takara; Prof Dennis Wenger; Dr Reid Basher; as well as Mr Jonathan Abrahams.

FUNDING INFORMATION

None to declare

CONFLICTS OF INTEREST

None to declare
